# Editorial: Mechanisms of hox-driven patterning and morphogenesis

**DOI:** 10.3389/fcell.2022.992341

**Published:** 2022-09-01

**Authors:** Marie Kmita, Edwina McGlinn, Ernesto Sánchez-Herrero

**Affiliations:** ^1^ Département de Médecine, Faculté de Médecine, Université de Montréal, Montreal, QC, Canada; ^2^ Genetics and Development Research Unit, Institut de Recherches Cliniques de Montréal, Montreal, QC, Canada; ^3^ Department of Experimental Medicine, McGill University, Montreal, QC, Canada; ^4^ Australian Regenerative Medicine Institute, Monash University, Clayton, VI, Australia; ^5^ Centre for Molecular Biology Severo Ochoa, Spanish National Research Council (CSIC), Madrid, Spain

**Keywords:** hox genes, drosophila, mouse, zebrafish, patterning, morphogenesis, evolution

The Hox genes have attracted the interest of scientist for decades. Their organization in genetic clusters, their ordered chromosomal alignment and the correlation of this disposition with the evolutionary conserved gene expression along the anteroposterior axis have excited the curiosity of researchers and prompted countless investigations. Hox proteins have also been studied as examples of transcription factors that activate particular genes at certain positions. The present Research Topic Mechanisms of Hox-Driven Patterning and Morphogenesis gathers a series of reviews and original articles on Hox expression and function in different model organisms.

The seminal work of Ed Lewis characterized transformations in the *Drosophila* cuticle due to mutations in Bithorax Complex (BX-C) genes. Subsequent studies revealed that Hox genes are expressed and required also in muscles or nervous system. Two reviews focus on the role of Hox genes in these tissues. The manuscript by Poliacikova et al. reviews the role of Hox genes in the different steps of muscle specification in *Drosophila* and vertebrates. The authors describe Hox activity in somatic/skeletal, cardiac and visceral muscle development, explain the cooperation of Hox proteins with muscle-specific proteins and explain the role of these genes in neuromuscular circuits. These neuromuscular networks are detailed in the work of Joshi et al. They report muscle-motoneuron circuits in *Drosophila* and how these impact on development and behavior. The authors first summarize Hox role in specification of the central nervous system and then review recent advances in Hox determination of motoneuron morphology and physiology, focusing in two larval behaviors, locomotion and feeding, and two adult ones, egg-laying and self-righting.

Although it is well established that Hox genes are required during development, recent studies have uncovered a Hox function in adults, as described in the above review. The original article by Li et al. demonstrates a role for Hox5 in adults. In a conditional triple mutant Hoxa5/Hoxb5/Hoxc5, in which Hox5 function is eliminated in the mesenchyme of adult mice, the investigators observed elimination of the elastin matrix, change in alveolar structures and an emphysema-like phenotype.

Two other research articles also unveil Hox5 function. In one of them (Mitchel et al.,) the activity of Hoxa5 in the development of the mouse sternum is analyzed. The authors first characterize in detail the origin and development of this structure. Then, they show Hoxa5 expression and requirement in the presternum, demonstrate the coordinated activity of other Hox genes in the formation of this structure and discuss the evolutionary implications of such function. In the second original article, by Howard et al., it is demonstrated that high levels of Hoxb5b in a restricted time window expand zebrafish neural crest cell number, increase the expression of the vagal neural crest cell markers *foxd3* and *phox2bb*, and extend the number of enteric neural progenitors; however, these progenitors do not expand later on to make enteric neurons along the gut. To explain the early expansion of neural crest cells but the later reduction of neurons, the authors argue that it can be due to the dynamic expression of Hox cofactors of the TALE family or to insufficiently timed signals needed for the continuous expansion and differentiation.

The most famous Hox mutation transforms halteres into wings in the fruitfly. Giraud et al. investigate the genetic basis of robustness and variation in haltere morphology, which is governed by the Hox gene *Ultrabithorax*. To this aim they carry out a genetic screen looking for haltere morphology changes in a wildtype or sensitized mutant background. Based on their results the authors postulate a self-sufficient mechanism whereby high levels of *Ultrabithorax* allow proper development of the haltere without the need for cofactors, thus ensuring its developmental stability.

The relationship between Hox gene function and evolution, originally proposed by Ed Lewis, is addressed in two reviews. In the first one, by Hombría et al., the authors describe the process of cephalization in vertebrates and arthropods and convincingly argue that the appearance of Hox cephalic genes predates the evolution of head structures in both groups of animals. As the formation of the cephalic region progressed during evolution, it incorporated trunk segments, and the Hox genes expressed in these segments acquired the control of specific genes to define cephalic structures. The second review (Krumlauf and Singh) deals with the role of Hox gene duplication and divergence in the development of morphology and in the emergence of new features in vertebrates. The authors discuss Hox gene duplication in evolution, showing examples of conservation and divergence of gene function, and explaining the role of the different domains of Hox proteins in the acquisition of specificity. They conclude that new specificity of Hox function can be achieved with just a few aminoacid changes in conserved regions and through interactions with proteins of the PBC class.

Cain and Gebelein discuss how the activity of different Hox proteins can determine the development of distinct structures by specific DNA binding and activation of particular genes. They summarize recent genomic and interactome data revealing how Hox proteins differ in the way they can bind closed chromatin, showing that some of them need PBC cofactors as pioneer factors. Then, they explain how the interaction of Hox proteins with cofactors and collaborators impinges on the way Hox proteins activate or repress gene expression, illustrating this differential Hox activity with the *Drosophila* Abdominal-A protein.

Finally, the comprehensive review by Hajirnis and Mishra discuss Hox organization and function. They first describe dispersion and clustering of Hox genes in different species, and then review the ordered disposition of cis-regulatory modules in the BX-C, and the opening of the BX-C chromatin, with emphasis on the organization of the *Abdominal-B* gene. They also review Hox function away from their traditional role of specifying particular structures and finally stress the importance of controlling Hox levels of expression by presenting examples of how increasing Hox protein levels can lead to cancer.

